# Is YouTube a reliable source of current information and education about urodynamics? A cross-sectional analysis

**DOI:** 10.1186/s12894-025-01983-5

**Published:** 2025-11-17

**Authors:** Murat Beyatlı, İsa Dağlı, Mehmet Duvarci, Oğuzhan Ceylan, Tuncel Uzel, Nurullah Hamidi, Erdem Öztürk

**Affiliations:** 1 Department of Urology, Ümraniye Training and Research Hospital, İstanbul, Turkey; 2 Department of Urology, Ankara Dr. Abdurrahman Yurtaslan Oncology Training and Research Hospital, Ankara, Turkey

**Keywords:** Youtube videos, Urodynamics, Source, Patient information, Discern

## Abstract

**Background:**

Urodynamic testing plays an important role in assessing lower urinary tract function. Due to the increasing use of the internet for medical information, platforms like YouTube have gained popularity as educational tools for medical procedures. However, the quality and reliability of this information are often uncertain. This study aims to evaluate the quality and reliability of YouTube videos as a source of current information and education on urodynamics.

**Methods:**

A search for “urodynamics” and “urodynamics test” on YouTube was conducted on March 8, 2024. The first 100 videos were screened, and 29 relevant videos were evaluated by two urologists. Videos were assessed for duration, number of views, likes, comments, and source, and scored for content quality using the British Association of Urological Surgeons (BAUS) criteria, Global Quality Score (GQS), and DISCERN tool.

**Results:**

Videos were categorized into “useful” and “non-useful” groups based on BAUS scores. While there was no significant difference in video popularity metrics between the groups, GQS and DISCERN scores were significantly higher in the “useful” group (*p* = 0.003 and *p* = 0.002, respectively). Videos from healthcare professionals, health information websites, and advertisements showed no significant differences in quality metrics, except for the number of subscribers, with advertisement channels having more subscribers (*p* = 0.026).

**Conclusion:**

YouTube contains useful information on urodynamics, but the overall quality varies. Efforts should be made to improve the content quality to better serve patients seeking information on this platform.

## Introduction

The primary functions of the lower urinary tract are the storage and voluntary emptying of urine. A healthy bladder stores urine at low pressure and enables controlled urination [1]. Urodynamic studies (UDS) are among the standardized tests used to evaluate lower urinary tract function and to diagnose underlying dysfunctions when initial evaluation is inconclusive [2, 3]. The purpose of urodynamic tests is to determine whether the bladder and other compartments of the lower urinary system are functioning correctly and to identify possible pathological conditions [1].

Before seeking medical attention, patients often extensively use the internet to understand their symptoms and to explore potential treatments. In particular, for interventional procedures, patients frequently watch online videos to learn about the process and overcome anxiety [4–6]. YouTube (http://www.youtube.com, Google LLC, California, United States) is one of the most popular social media platforms worldwide and is commonly used for this purpose. ^6^.

Previous studies have examined the content and quality of YouTube videos related to prostate biopsy, premature ejaculation, and transdermal testosterone therapy [7–9]. These studies have highlighted the variable reliability and educational value of YouTube content in urology.

However, there is currently no research evaluating videos related to UDS on YouTube. Therefore, the aim of this study was to assess the quality and reliability of YouTube videos as a source of information and education for patients on UDS.

## Materials and methods

This study was conducted as a cross-sectional analysis. In accordance with our institution’s policy, studies based solely on publicly available data and without involvement of human subjects do not require ethics committee review.

The primary endpoint was the comparison between videos classified as ‘useful’ and those classified as ‘non-useful’ with respect to their characteristics and quality scores. The secondary endpoints were analyses according to video source (healthcare professionals, health information websites, advertisements).

### Search strategy

Videos available on YouTube (http://www.youtube.com*)* related to the keywords " urodynamics” and " urodynamics test " were searched on March 8, 2024, using the video search engine. The videos were searched according to the automatic settings on the internet platform. Previous viewing history and personal search records may significantly influence the YouTube algorithm and retrieved results. To minimize this effect, all searches were performed using the browser’s “incognito mode” and additionally repeated through a newly created YouTube account without any prior activity. Furthermore, to reduce potential country-based algorithmic bias, a VPN was used to connect with a United Kingdom IP address, and searches were performed from this location. This combined approach was intended to provide a more neutral and reproducible video list, independent of both personalized recommendations and geographical variations. As most people tend to watch videos on the first pages of search results, the first 100 videos were saved by creating a new playlist. All videos were evaluated separately by two urologists. Videos that were longer than 1 min but shorter than 10 min and in the English language were included in the evaluation. This duration range was selected because videos shorter than 1 min (often including “Shorts”) are unlikely to provide sufficient educational content, whereas videos exceeding 10 min may reduce viewer attention and engagement. Videos that were not in a single piece and repetitive videos were not included in the study. Videos without sound or unrelated to the subject were excluded. A total of 29 videos meeting these criteria were recorded. Videos that were fragmented or repetitive were excluded from the study.

### Data collection

Video Characteristics and Scoring System; The selected videos were analyzed for duration, number of likes, number of views, daily views, number of comments, initial release date, and video source data. Based on their sources, the videos were divided into 3 groups according to source: healthcare professionals, health information website and advertisement. There is no standardized analysis system for evaluating YouTube videos, but there are generally accepted methods in the literature. In this study, we used three different scoring systems to measure the content and quality of the videos.

**Fig. 1:**
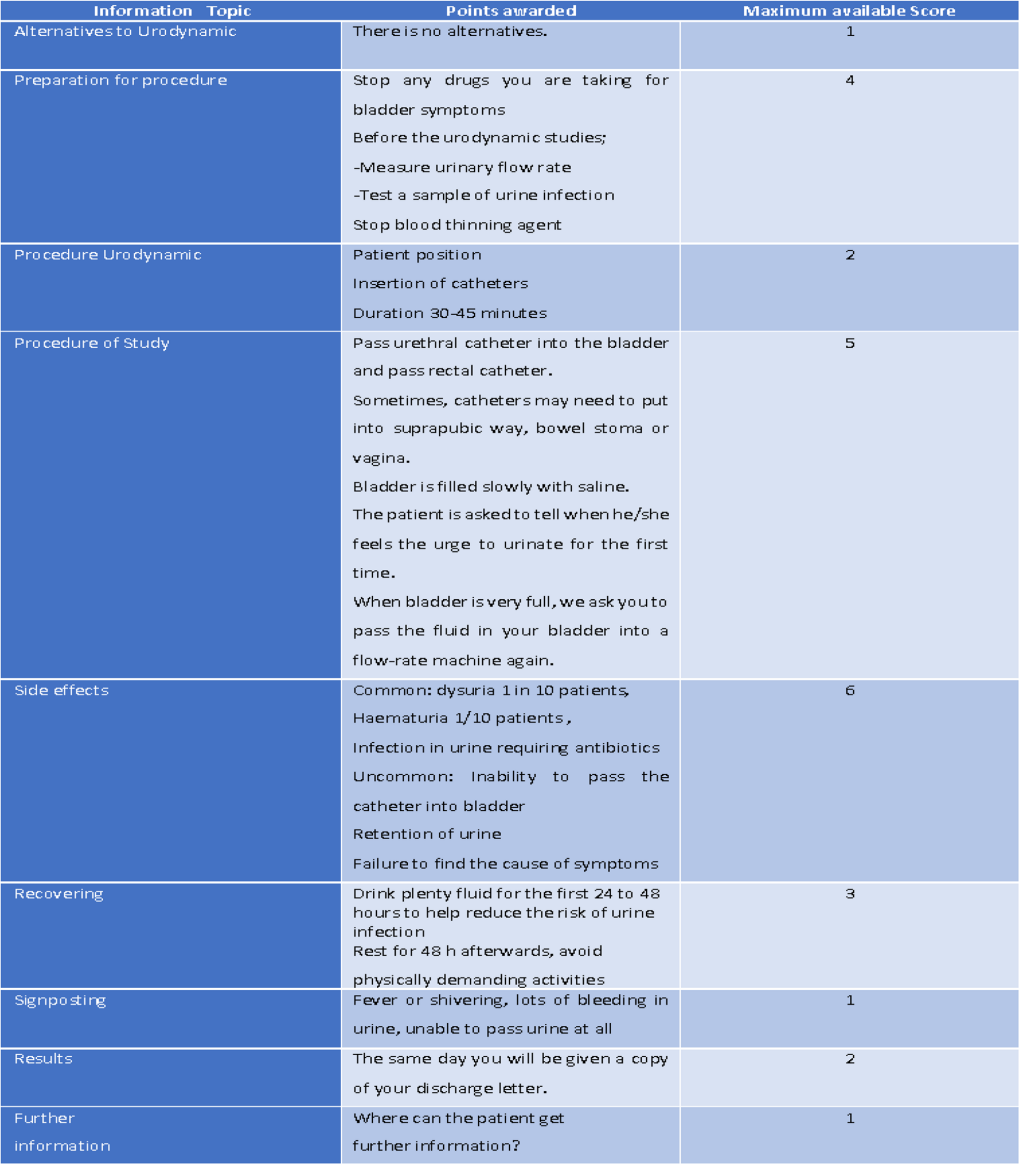
The British Association of Urological Surgeons (BAUS) based video scoring system

To evaluate urodynamics, we used the brochure provided by the British Association of Urological Surgeons (BAUS). This brochure has an evidence-based and comprehensive scoring system. The brochure consists of a total of 9 sections and is scored out of a total of 25 points. Accordingly, videos that scored above 10 points were classified as reliable, while those scoring 10 points or below were considered unreliable [10]. The quality assessment of the individuals rating the videos is categorized as “very poor” (0–5), “poor” (6–10), “acceptable” (11–15), “good” (16–20), and “excellent” (21–25) (Fig. 1).

**Fig. 2:**
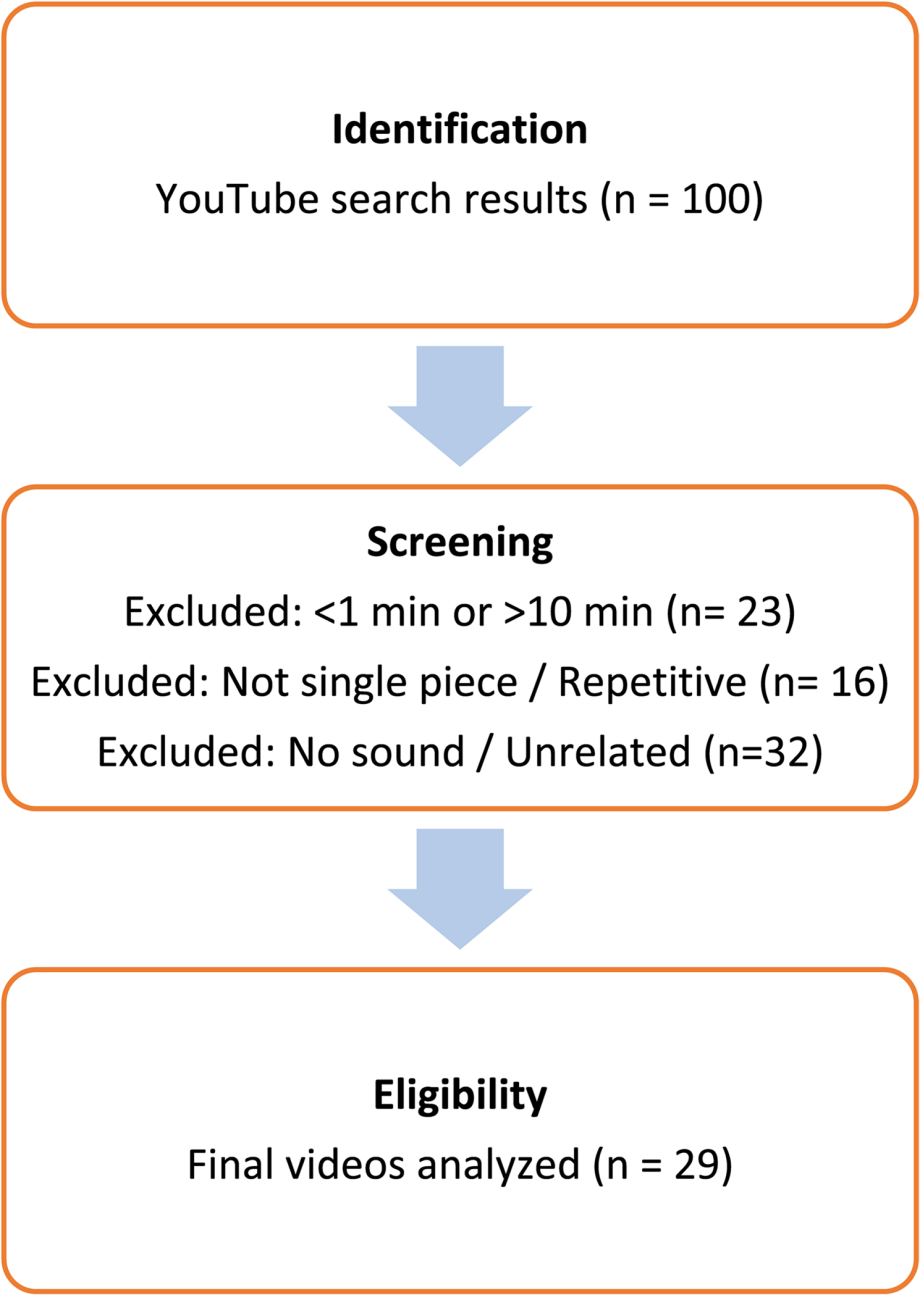
Flow chart of the video selection process

The quality of the videos was scored using the Global Quality Score (GQS) form. GQS was developed to evaluate web resources based on the quality of information and how useful a specific video would be for a patient, as assessed by the reviewer [11]. In GQS, 1 point is considered low quality with very little likelihood of benefit to patients, while 5 points are considered excellent quality and flow, with a high degree of benefit to patients (Fig. 2).

We used the DISCERN, a validated tool consisting of 15 questions rated on a 5-point scale, developed by Charnock and colleagues to assess the quality of health information presented to patients regarding treatment options [12].

### Statistical analysis

All statistical analysis was done by using SPSS 25.0 (IBM, Chicago, IL, USA). Shapiro-Wilk test was used to control the normality of variables. Mann-Whitney U and Kruskal-Wallis tests were used to evaluate the differences between groups. Bonferroni post-hoc correction was used after the Kruskal-Wallis test. For statistical significance, p value of < 0.05 was accepted.

## Results

The videos were categorized into “useful” and “non-useful” groups based on BAUS criteria. Total views, video length, duration on Youtube, likes, views per day, comments, and number of subscribers were evaluated in both groups. No statistical significance was observed in these categories. The mean GQS score for useful videos was 4.66 ± 0.51 compared to 3.17 ± 1.07 for non-useful videos, while the mean DISCERN score was 4.16 ± 0.40 versus 2.56 ± 1.16, respectively. The GQS and DISCERN scores were higher in the “useful” group, and this difference was found to be statistically significant (*p* = 0.003 and *p* = 0.002, respectively). Video characteristics by non-useful category were shown in Table-1.

**Table 1:** Video characteristics by usefulness category

	Useful information	Non-useful information	*P* value
	n:6	n:23	
Total views	23,983 (4903,50–33214,50)	5600 (1168–17343)	0.306^δ^
Video Length (s)	294,50 (203,25–383)	215 (129–320)	0.236^δ^
Duration on YouTube (months)	62,65 (24,23–70,05)	52 (16–110,40)	0.914^δ^
Likes	119 (49–181,5)	19 (6–63)	0.131^δ^
Views per day	13,61 (7,22 − 21,17)	3,88 (2,24 − 11,28)	0.178^δ^
Comments	16,83 ± 26,82	12,13 ± 36,31	0.063^δ^
Number of subscribers	3394 (173,75–19475)	4460 (198–33300)	0.647^δ^
GQS Score	4,66 ± 0,51	3,17 ± 1,07	**0.003** ^δ^
Discern Score	4,16 ± 0,40	2,56 ± 1,16	**0.002** ^δ^

^**δ**^Mann *Whitney-U test*

*Bold values indicate the signifcant of p < 0.05*

*Variables are presented as median value(Q1–Q3) and mean ± standart deviation*

Videos were further categorized by their sources into three groups: healthcare professionals, health information websites, and advertisements. Upon analysis, no statistical significance was found in Total views, video length, duration on Youtube, likes, views per day, comments, GQS, and DISCERN scores among these categories. Only the number of subscribers were found to be statistically significant (*p* = 0.026). Video characteristics by source were shown in Table-2. Pairwise analysis of the number of subscribers between the groups revealed that advertisement channels had significantly more subscribers compared to healthcare professionals, and this difference was statistically significant (*p* = 0.036). Pairwise comparison of video sources were shown in Table-3.

**Table 2:** Video characteristics by source

	Healthcare professionals(*n*:17)	Health information(*n*:4)	Advertisement(*n*:8)	*P* value
Total views	11,035 (3684–19154,50)	171 (122,50–56298,50)	19,300 (1384,75–62978,25)	0.218^ε^
Video Length (s)	287 (163–371)	259 (12,25–402)	196 − 50 (135–258)	0.439^ε^
Duration on YouTube (months)	68,50 (29,72–117,73)	26,58 (8,18–49,58)	49,61 (15,67–86,19)	0.138^ε^
Likes	29 (7–104)	1,50 (0,25–244,25)	34,50 (8–188,75)	0.282^ε^
Views per day	7,91 (2,65 − 12,57)	0,34 (0,16–38,64)	8,92 (2,80 − 43,24)	0.235^ε^
Comments	11,52 ± 20,60	1,25 ± 2,50	22,37 ± 59,28	0.267^ε^
Number of subscribers	484 (151,50–11180)	166,50 (61,ID="EN9">25–34252)	23,350 (9435–42625)	**0.026** ^ε^
GQS Score	3,35 ± 1,16	3,25 ± 1,50	3,87 ± 0,99	0.535^ε^
Discern Score	2,82 ± 1,38	3,25 ± 1,25	2,87 ± 0,99	0.930^ε^

^ε^Kruskal Wallis test

*Bold values indicate the signifcant of p* < 0.05

*Variables are presented as median value(Q1–Q3) and mean ± standart deviation.*

**Table 3:** Pairwise comparison of video sources

	*p* values			
	Health information website vs. Healthcare professionals	Health information website vs. Advertisement	Healthcare professionals vs. Advertisement	
Number of subscribers	0.999	0.111		**0.036**

Kruskal Wallis test

*Bold values indicate the significance of p* < 0.05

*Significance values have been adjusted by the Bonferroni correction for multiple tests*

## Discussion

In this study, we evaluated the quality and reliability of urodynamic videos on YouTube. Our results revealed that although YouTube contains useful information about urodynamics, its overall quality varies.

As a result of the increasing frequency of Internet use, patients’ use of social media platforms to access medical information has steadily increased [13, 14].YouTube is one of the most popular video platforms worldwide. It has become an important source for patients to learn about medical procedures [15].

When examining YouTube, it becomes evident that there is a wealth of practical and theoretical information on various medical topics [16, 17]. However, the quality and reliability of videos on urodynamics have not been thoroughly researched. To the best of our knowledge, our study is the first study to question the quality and reliability of urodynamic videos on Youtube. In our study, we analyzed the quality of the first 100 YouTube videos related to urodynamics. When we examined the first 100 videos that appeared when searching for “urodynamics” on YouTube, we found that 71 of these videos were either irrelevant or contained promotional content such as device demonstrations. Some videos were excessively long, potentially causing the patient’s attention to wander. Out of the first 100 videos, only 29 contained informative content about urodynamics.

From the 29 videos included in the study, we categorized those with a BAUS score above 10 as the useful group and those with a BAUS score below 10 as the non-useful group.

In our study, the number of videos categorised as useful according to BAUS criteria (*n* = 6) was significantly lower than the number of videos that were not useful (*n* = 23). These findings showed that quality content on urodynamics on YouTube is limited. It also suggests that patients may have difficulty accessing reliable information. It was also reported in the study by Osman et al. that the quality of health information on YouTube was generally inadequate and did not meet standard quality criteria [6].

No significant correlation was found between the number of views, number of likes, number of comments and video quality. This showed that patients may have difficulty in recognising quality content. It also shows that popular videos do not necessarily contain accurate information.In the literature review by Afful-Dadzie et al. it was reported that there was an inconsistent relationship between popularity metrics and quality of health information on social media platforms [15]. This suggests that patients may struggle to identify high-quality content.

In terms of GQS and DISCERN scores, useful videos received significantly higher scores than nonuseful videos. Our findings showed that evaluation tools such as GQS and DISCERN were effective in objectively assessing the quality of YouTube videos. The DISCERN tool has been previously confirmed in many studies to be a reliable tool for assessing the quality of health information [12, 18]. Similarly, the GQS has been shown to be a widely used and reliable tool for assessing medical video quality [19]. From this, we can conclude that while patients are successful in accessing more useful information, the content of the videos is often inadequate.

It was seen that most of the videos analysed for evaluation in the study were irrelevant to the subject or were promotional videos. This situation suggested that there was more content prepared with commercial concerns. In the systematic review conducted by Madathil et al. it was stated that commercialisation of health information on YouTube is an important concern [20].

One of the limitations of our study can be considered as selecting only among the first 100 videos and being limited to 29 videos. The first 100 videos evaluated may not represent all available content [21]. At the same time, the fact that only 6 of the 29 videos are in the useful group may be insufficient to produce statistical significance due to the small sample size. In addition, our study only evaluated videos in English and content in other languages was not included in the study. Another limitation of our study is that the ranking position of each video (i.e., page number and order within the page) in the YouTube search results was not recorded. As a result, we were unable to analyze the accessibility of the “useful” videos in relation to their placement within the search results. This information would have provided additional insights into how easily patients may encounter reliable content.

To increase patients access to reliable information, healthcare professionals should take a more active role on platforms such as YouTube. In a systematic review by Helming et al. it was emphasised that standard criteria and peer review processes should be implemented to improve the quality of YouTube videos for medical education [22]. In addition, medical associations and academic institutions should take a more active role in the production of standardized and reliable content [23].

It is important to disseminate health literacy programmes to improve patients’ ability to evaluate health information. In a study by Diviani et al. it was shown that individuals with low levels of health literacy had more difficulty in evaluating online health information [24]. Digital health literacy has become an integral part of the modern healthcare system and is necessary to ensure that patients have access to accurate information from reliable sources [25].

Finally, although it is known that video-based materials are effective in patient education, the quality of these materials should be standardized and updated regularly.

## Conclusion

YouTube contains useful information about urodynamics, its overall quality standards are inadequate. The lack of a significant correlation between popularity measures and content quality suggests that patients may have difficulty in recognising quality content. Efforts should be made for health professionals and medical institutions to take a more active role in social media platforms, to produce evidence-based content and to improve patients’ digital health literacy skills. Improving the quality of information on these platforms is critical for patient education and improving health outcomes. It is recommended that regular quality controls should be carried out using standardised assessment tools and health professionals should take a more active role in social media platforms. Although YouTube contains useful information about urodynamics, the overall quality is inadequate.

## Data Availability

The datasets used and/or analyzed during the current study are available from the corresponding author upon reasonable request.

## Abbreviations

BAUS: British Association of Urological Surgeons
GQS: Global Quality Score
UDS: Urodynamic Studies
